# PReS-FINAL-2307: Libman-Sacks endocarditis as a presentation for systemic lupus erythematous in an adolescent with isolated mitrial regurgitation and Noonan syndrome

**DOI:** 10.1186/1546-0096-11-S2-P297

**Published:** 2013-12-05

**Authors:** SC Chen, B Smith, M Ilina, C Bowen, N Martin

**Affiliations:** 1 Paediatrics, Royal Hospital for Sick Children, Glasgow, UK; 2 Cardiology, Royal Hospital for Sick Children, Glasgow, UK; 3 Pathology, Royal Hospital for Sick Children, Glasgow, UK; 4 Rheumatology, Royal Hospital for Sick Children, Glasgow, UK

## Introduction

Systemic lupus erythematous (SLE) is a complex disease which is rare in childhood and can present insidiously. Libman-Sacks endocarditis (LSE) is a recognised cardiac manifestation of SLE with valvular abnormalities that are clinically silent prior to significant valve dysfunction. Multiple reports have associated isolated mitral regurgitation with SLE and the presence of antiphospholipid antibodies in SLE patients increases the prevalence of mitral valve regurgitation by three fold.

## Objectives

To highlight awareness of Libman-Sacks endocarditis as a presentation of juvenile SLE

## Methods

Case report

## Results

We present a case of a 17 year old boy with phenotypic Noonans (SHOC2 mutation) who presented aged 14 with a pericardial effusion, verging on tamponade and requiring surgical drainage and diuretic therapy. A previous cardiac ultrasound aged 12 had shown no significant abnormality. One year later he developed mitral regurgitation, deteriorating over the following 18 months with the development of increasingly severe congestive cardiac failure (WHO class IV). Further history revealed new onset headaches and difficulty in concentration as well as a history of intermittent arthralgia. There were no rash, fever or mouth ulcers.

Investigations demonstrated lymphopenia, prolonged APTT with positive lupus anticoagulant and anticardiolipin antibodies, raised immunoglobulin levels, low C4, persistently raised ESR but normal CRP as well as strongly positive ANA(1:2560) and anti-DNA antibody(86 IU/ml). Blood cultures, throat swabs and viral serology were negative. A diagnosis of SLE with Libman-Sacks endocarditis was made. There was no evidence of renal involvement. A course of intravenous methylprednisolone followed by oral steroids was given to minimise active inflammation prior to mitral valve replacement with a mechanical valve. Histopathology of the damaged mitral valve demonstrated fibrinous deposits with neovascularisation and myxoid degenerative changes, consistent with LSE. He made a good recovery following surgery with resolution of his dyspnoea. He has been anticoagulated with warfarin and commenced on hydroxychloroquine and mycophenolate mofetil for maintenance immunosuppression.

## Conclusion

LSE is rare in childhood with only six previous cases described in the literature. In adults with SLE the prevalence of progressive valvular abnormalities is higher when SLE is associated with antiphospholipid antibodies. Interestingly, SHOC2 mutation is associated with congenital mitral valve defects but acquired mitral valve disease has not been reported.

This case highlights the difficulties and potential delays in diagnosing SLE due to its varied and often insiduous presentation and demonstrates that LSE can occur in children with lupus. It also reaffirms the importance of considering autoimmune inflammatory conditions in cases of pericarditis with no evidence of an infectious cause.

## Disclosure of interest

None declared.

